# Causal associations between schizophrenia and cancers risk: a Mendelian randomization study

**DOI:** 10.3389/fonc.2023.1258015

**Published:** 2023-11-17

**Authors:** Kai Zhou, Lin Zhu, Nian Chen, Gang Huang, Guangyong Feng, Qian Wu, Xiao Wei, Xiaoxia Gou

**Affiliations:** ^1^ Department of Head and Neck Oncology, The Second Affiliated Hospital of Zunyi Medical University, Zunyi, Guizhou, China; ^2^ Department of Oncology, The People’s Hospital of Linshui, The Second Affiliated Hospital, Chongqing Medical University, Chongqing, China

**Keywords:** cancer, schizophrenia, Mendelian randomization, causal association, TSMR

## Abstract

**Background:**

Previous observational studies have reported inconsistent findings regarding the incidence of cancer in patients with schizophrenia compared to the general population. The causal relationship between schizophrenia and cancer remains unclear and requires further investigation.

**Objective:**

To investigate the causal relationship between schizophrenia and cancer.

**Methods:**

In this study, a two-sample Mendelian randomization (MR) analysis was conducted using publicly available genome-wide association studies to determine the causal relationship. The effect estimates were calculated using the random-effects inverse-variance-weighted method.

**Results:**

We determined a causal relationship between genetic predisposition to schizophrenia and cancer, with schizophrenia increasing lung cancer (odds ratio (OR) = 1.0007; 95% confidence interval (CI), 1.0001-1.0013; *p* = 0.0192), thyroid cancer (OR = 1.5482; CI, 1.1112-2.1569; *p* =0.0098),colorectal cancer (OR = 1.0009; CI, 1.0001-1.0018; *p* = 0.0344), ovarian cancer (OR = 1.0770; CI, 1.0352-1.1203; *p* = 0.0002), breast cancer (OR = 1.0011; CI, 1.0001- 1.0022; *p* =0.0352) and reduced the risk of malignant neoplasm of the stomach (OR = 0.8502; CI, 0.7230-0.9998; *p* = 0.0496).

**Conclusions:**

This study conducted a two-sample MR analysis and discovered a positive causal relationship between schizophrenia and breast, ovarian, thyroid, lung, and colorectal cancers. On the other hand, an inverse causal relationship was found between schizophrenia and malignant neoplasm of the stomach.

## 1 Introduction

Cancer is a major cause of death worldwide and a significant obstacle to increasing life expectancy in all countries (1). According to projections, the United States is expected to see 1,918,030 new cases of cancer and 609,360 cancer-related deaths in 2022. Lung cancer is the primary cause of cancer deaths, with approximately 350 deaths occurring daily (2). The global cancer burden is projected to increase to 28.4 million cases by 2040, a 47% rise from 2020. This increase is expected to be larger in transitioning (64% to 95%) versus transitioned (32% to 56%) countries due to demographic changes, and may be further exacerbated by increasing risk factors associated with globalization and a growing economy (3). Cancer has several common risk factors, including an unhealthy diet, physical inactivity, smoking, advanced age, and family history. However, in order to further reduce the burden of cancer, it is important to also consider other potentially modifiable risk factors, such as psychiatric conditions (4).

The relationship between psychiatric disorders and tumors has been a topic of interest and controversy in the medical community (5). Some epidemiological studies and meta-analyses have suggested a potential link between schizophrenia and an increased risk of breast cancer (6, 7). Previous research and meta-analyses have demonstrated that individuals with schizophrenia have a heightened risk of cancer-related mortality, with a 50% increase in comparison to their healthy counterparts of the same gender and age (8, 9). Establishing a causal relationship between exposure and outcome in observational studies is a challenging task. While randomized controlled clinical trials are considered the gold standard, their implementation may be hindered by various challenges such as high costs and time constraints. Hence, it is crucial to adopt a simple yet effective approach to investigate the causal link between schizophrenia and cancer.

In recent years, the use of large-scale genome-wide association studies (GWAS) and Mendelian Randomization (MR) research has become increasingly popular in investigating the causal relationship between complex exposure factors and disease outcomes. This is due to the availability of better statistical methods and high-throughput sequencing technology (10). In the context of Mendelian randomization (MR), genetic variants are employed as instrumental variables (IVs) to explore the potential causal relationship between environmental exposures (such as schizophrenia) and outcomes (such as cancer) (11). The design has two advantages. Firstly, it can minimize confounding effects as genetic variants are randomly assorted during conception and hence not correlated with environmental or self-adopted factors that usually act as confounders in the association between exposure and outcome (12). Secondly, this approach can reduce the possibility of reverse causality since the onset and progression of disease cannot modify the germline genotype (12).

Previous MR studies have shown a positive association between schizophrenia and the risk of ovarian, thyroid and breast cancers (13). However, in contrast to clinical observations, patients with schizophrenia may be at greater risk for other cancers, such as lung cancer. Here, we conducted an MR investigation to explore the causal relationship of these associations. In this study, we investigated the causal relationship between schizophrenia and a wide range of cancers by conducting a comprehensive two-sample MR analysis of 11 cancers from the IEU Open GWAS program, including breast, colorectal, ovarian, head and neck, lung, hepatocellular, thyroid, prostate, esophageal, gastric malignancies, and pancreatic cancers. This study aims to establish a causal link between schizophrenia and cancer to aid in the prevention of cancer development in patients with schizophrenia.

## 2 Materials and methods

Ethics approval was not necessary for this analysis as all the GWAS data used were publicly available and had already been approved by the respective ethical review boards.

### 2.1 Study design

**Figure 1:**
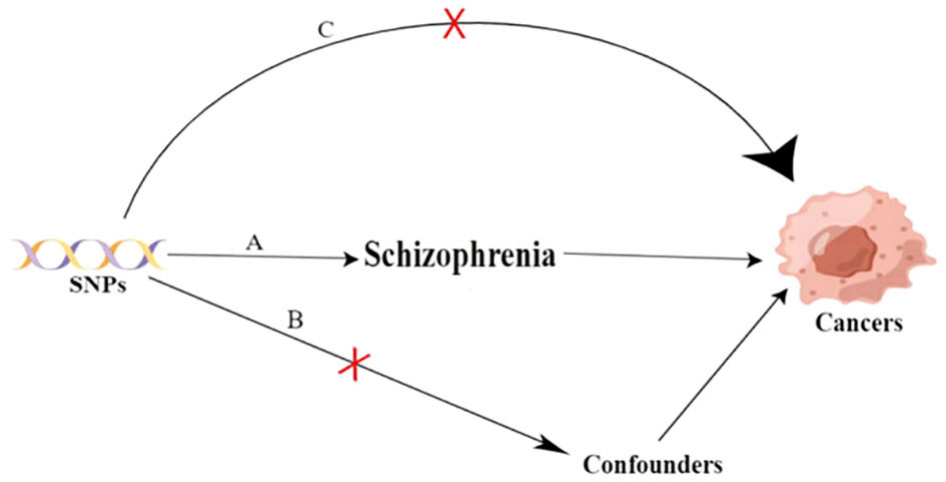
Three key assumptions of the Mendilian randomization study. A. Strong association of single nucleotide polymorphisms (SNPs) with schizophrenia; B. Independence of SNPs from known confounders; C. SNPs solely influence cancer through schizophrenia. SNP: single-nucleotide polymorphism.

The study design and three key assumptions of MR are depicted in **Figure 1**.

### 2.2 Data source

#### 2.2.1 Exposure data

The SNPs chosen as instrumental variables were obtained from a two-stage genome-wide association study that involved 76,755 patients with schizophrenia and 243,649 control patients. The study identified common variant associations at 287 different genomic loci (14). Phase I genome-wide association study, the primary GWAS, including 74,776 patients with schizophrenia and 101,023 controls; Data from a core Psychiatric Genomics Consortium (PGC) dataset of 90 cohorts of European (EUR) and East Asian (ASN) ancestry from the PGC, totalling67,390 cases and 94,015 controls; and summary-level data from 7,386 cases and 7,008 controls from 9 cohorts of African American (AA) and Latino (LAT) ancestry. This study analyzed up to 7,585,078 single-nucleotide polymorphisms (SNPs) with a minor allele frequency (MAF) greater than or equal to 1% in 175,799 individuals of whom 74.3% were EUR, 17.5% ASN, 5.7% AA and 2.5% LAT. This primary GWAS identified 313 independent SNPs (linkage disequilibrium (LD) r^2^ < 0.1) that exceeded genome-wide significance (*p* < 5 × 10^−8^), spanning 263 distinct loci (15). The second phase of the genome-wide association study, the extended GWAS, A meta-analysis of the primary GWAS results using summary statistics from deCODE genetics (1,979 cases, 142,626 controls) for index SNPs with *p* < 10−5 and identified 342 linkage-disequilibrium-independent significant SNPs located in 287 loci (16).

#### 2.2.2 Outcome data

We utilized publicly available data from the MRC Integrative Epidemiology Unit (IEU) cancers GWAS summary data (accessed on May 6th, 2023) to conduct our analysis. The GWASs of interest were conducted on predominantly European individuals and included both males and females, and we used published summary-level data from these studies. Lung cancer (*n* = 374,687), liver cell carcinoma (*n* =372,184), prostate cancer (*n* =182,625), esophageal cancer(*n* =372,756), head and neck cancer(n= 373,122) was obtained from UK Biobank Consortium. Thyroid cancer (*n* = 1,080) was obtained from the results reported by Köhler A, et al. (17). Ovarian cancer (*n* = 66,450) was obtained from the Ovarian Cancer Association Consortium (OCAC) (18). Liver cell carcinoma (*n* =372,184), breast cancer (n=337,159) was obtained from Neale Lab Consortium. Malignant neoplasm of stomach (*n* = 218,792) were obtained from FinnGen Consortium. Pancreatic cancer (*n* = 3,835) was obtained from the results reported by Amundadottir L, et al. (19).

### 2.3 Instrumental variable selection

To ensure the accuracy and authenticity of the causal link between schizophrenia and cancer risk, we employed quality control measures to select the optimal instrumental variables (IVs). We first selected SNPs that were significantly related to schizophrenia as IVs, using two thresholds. The first threshold selected SNPs below the genome-wide statistical significance threshold (5 × 10^−8^) as IVs. Secondly, we ensured that there was no linkage disequilibrium (LD) among the included IVs, as the presence of strong LD could result in biased results. To assess the LD between the included SNPs, we conducted the clumping process (R^2^ < 0.001 and clumping distance =10,000 kb). Third, to avoid distortion of strand orientation or allele coding, we deleted palindromic SNPs. Fourth, by searching in Phenoscanner (http://www.phenoscanner.medschl.cam.ac.uk/, a database of over 65 billion linked genetic variants and over 150 million unique results from large-scale global warming studies), we eliminated SNPs associated with cancer etiology.

### 2.4 MR analysis

In this study, we conducted an MR analysis to explore the causal relationship between schizophrenia and common cancers. To analyze features containing multiple IVs, we employed five popular MR methods, namely inverse-variance weighted (IVW) test (12), weighted mode (20), MR-Egger regression (21), weighted median estimator (WME) (22), and MR-PRESSO (23). Although the IVW method is considered to be slightly more powerful than the other methods under certain conditions (22), we used all five methods in our analysis. However, the results of the study were mainly based on the IVW method, with the other four methods serving as complementary analyses.

As a sensitivity analysis, two methods - weighted median and MR-Egger were employed. The weighted-median method is considered valid if more than 50% of the information is derived from valid IVs (24). The MR-Egger method, on the other hand, is useful in evaluating the horizontal pleiotropy of selected IVs (21). Cochrane’s Q-value can indicate heterogeneity among selected IVs. A Q larger than the number of instruments minus one provides evidence for heterogeneity and invalid instruments, or Q statistics significant at a *p* < 0.05 can imply the presence of heterogeneity (25, 26). To evaluate the reliability of the findings, various sensitivity analyses were conducted. A leave-one-out analysis was performed to determine if a single SNP was responsible for the causal signal. This method compares the variance explained by the instrumental variables (IVs) for both the exposure and outcome. If the IVs explain a greater variance in the exposure than in the outcome, then the causal association identified can be considered directionally credible (27).

## 3 Results

### 3.1 SNP selection

The studies analyzed in this research were published from 2009 to 2022 and focused primarily on the European population (**Supplementary Table S1**). By going below the genome-wide statistical significance threshold (5 × 10−8), removing LD (R2 < 0.001 and clumping distance =10,000 kb), and deleting palindromic SNPs, we obtained 217 SNPs strongly associated with schizophrenia. Subsequently, we removed 11 (rs2970610, rs12126806, rs2139054, rs778371, rs7582445, rs7604885, rs1604060, rs1866862, rs2710323, rs13107325, and rs140365013) SNPs that were closely associated with cancer by Phenoscanner screening. Finally, a total of 206 independent variables that achieved a genome-wide significance level were chosen for analysis (**Supplementary Table S2**).

### 3.2 Cancers

**Figure 2:**
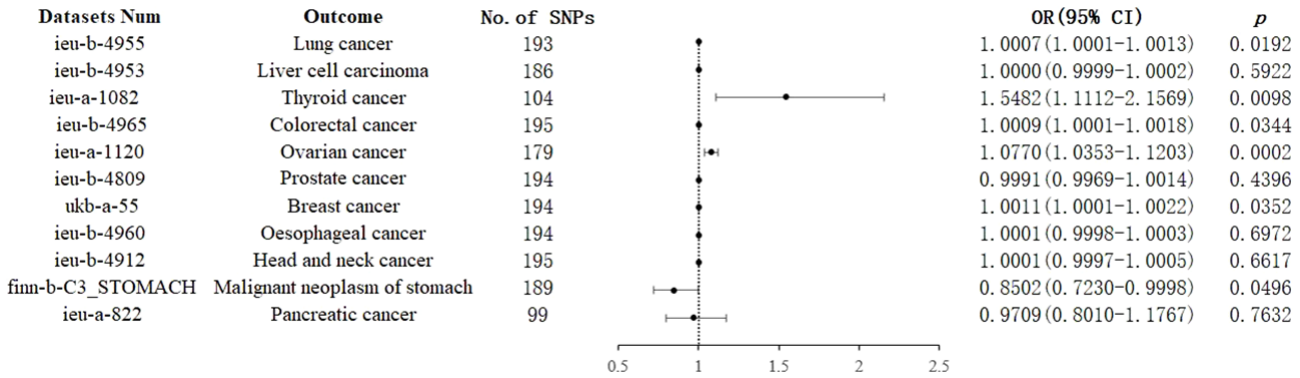
Associations of genetically predicted schizophrenia with cancers. CI, confidence interval; OR, odds ratio; SNP, single-nucleotide polymorphism.

IVW analysis showed that genetically predicted increases in schizophrenia per standard deviation were positively associated with 5 of 11 cancers and 1 was negatively associated, including, with increasing or decreasing magnitude of association, lung cancer (odds ratio (OR) = 1.0007; 95% confidence interval (CI), 1.0001-1.0013; *p* = 0.0192), thyroid cancer (OR = 1.5482; CI, 1.1112-2.1569; *p* = 0.0098),colorectal cancer (OR = 1.0009; CI, 1.0001-1.0018; *p* = 0.0344),ovarian cancer (OR = 1.0770; CI, 1.0352-1.1203; *p* = 0.0002), breast cancer (OR = 1.0011; CI, 1.0001-1.0022; *p* =0.0352) and malignant neoplasm of stomach(OR = 0.8502;CI, 0.7230-0.9998; *p* = 0.0496). In contrast, no associations were observed for liver cell carcinoma (OR = 1.0000; CI, 0.9999-1.0002; *p* = 0.5922), head and neck cancer (OR = 1.0001; CI,0.9997-1.0005; *p* =0.6617), prostate cancer (OR=0.9991; CI,0.9969-1.0014; *p* =0.4396), esophageal cancer (OR=1.0001; CI, 0.9998-1.0003; *p* =0.6972), and pancreatic cancer (OR=0.9709; CI, 0.8010-1.1767; *p* =0.7632) (**Figure 2**). For most cancers, the weighted-median and MR-Egger analyses revealed consistent estimates but of low precision (**Table 1**). No evidence of directional pleiotropy was detected. The heterogeneity was higher for some cardiovascular diseases. Therefore, an IVW meta-analysis under a random-effects model was adopted to mitigate the influence of heterogeneity.

**Table 1:** Associations between genetically predicted schizophrenia and eleven cancers in sensitivity analyses using the weighted-median and MR-Egger methods.

Outcome	Weighted Median		MR-Egger		Pleiotropy		Heterogeneity	
	OR (95% CI)	*p*	OR (95% CI)	*p*	Intercept	*p*	Q	*p*
Lung cancer	1.0007(0.9999- 1.0016)	0.0560	0.9995(0.9971- 1.0019)	0.6873	7.06E-05	0.3233	212.3767	0.1494
Liver cell carcinoma	1.0001(0.9999- 1.0003)	0.5785	0.9996(0.9988- 1.0003)	0.2588	2.78E-05	0.2067	217.3450	0.0519
Thyroid cancer	1.6925(1.0596- 2.7032)	0.0276	2.2648(0.3758-13.6474)	0.3744	-2.17E-02	0.6736	109.1004	0.3216
Colorectal cancer	1.0009(0.9998- 1.0021)	0.1155	0.9993(0.9957- 1.0030)	0.7320	9.52E-05	0.3822	245.9994	0.0068
Ovarian cancer	1.0922(1.0295- 1.1586)	0.0034	1.0818(0.9018- 1.2978)	0.3982	-2.67 E-04	0.9603	173.1907	0.5878
Prostate cancer	0.9999(0.9969- 1.0030)	0.9712	0.9965(0.9866- 1.0066)	0.4963	1.55 E-04	0.6017	235.9791	0.0189
Breast cancer	1.0012(0.9998- 1.0027)	0.0968	0.9993 (0.9946- 1.0039)	0.7598	1.11 E-04	0.4272	215.9591	0.1233
Oesophageal cancer	0.9998(0.9993-1.0002)	0.2623	0.9995(0.9982-1.0008)	0.4916	3.05E-05	0.4271	192.9608	0.4873
Head and neck cancer	1.0004(0.9999-1.010)	0.1009	0.9995(0.9979-1.0011)	0.5832	3.25E-05	0.5010	238.4424	0.0163
Malignant neoplasm of stomach	0.8408(0.6623-1.0675)	0.1546	1.2390(0.6163-2.4912)	0.5482	-2.25 E-02	0.2786	189.1726	0.4623
Pancreatic cancer	0.9184(0.6959- 1.2121)	0.5477	2.6274(1.0598- 6.5136)	0.0397	-5.73 E-02	0.0304	101.5421	0.3830

CI, confidence interval; MR, Mendelian randomization; OR, odds ratio.

**Supplementary Figures S1**, **S2** display scatter plot and forest plot, respectively, showcasing the association between schizophrenia and eleven cancers. The results are similar in both plots. **Supplementary Figure S3** shows the leave-one-out sensitivity analysis, indicating that no individual SNP disproportionately affected the overall estimates. The funnel plot in **Supplementary Figure S4** suggests no evidence of horizontal pleiotropy.

## 4 Discussion

For the causal relationship between schizophrenia and common cancers, we performed a two-sample MR analysis. Our findings showed that schizophrenia was positively associated with lung, thyroid, colorectal, ovarian, and breast cancers, and negatively associated with malignant neoplasm of the stomach.

Compared to the general population, published studies have revealed increased, decreased, or similar cancer incidence in patients with schizophrenia. However, the relationship between cancer and schizophrenia is not simple and may even be contradictory (9). Based on a meta-analysis of 16 cohort studies with a total of 480,356 participants diagnosed with schizophrenia and 41,999 cases of cancer, the results indicate a decreased overall risk of cancer incidence among patients with schizophrenia (RR=0.90, 95% CI 0.81-0.99) (28). In a recent study conducted by Momen et al., based on Danish data that included 5.9 million individuals, a slightly increased risk of being diagnosed with cancer for the first time after the diagnosis of schizophrenia was identified. The hazard ratio was 1.05 (1.03-1.08) (29). A study conducted on Swedish register-based data found that individuals with schizophrenia did not exhibit a higher incidence of cancer compared to the general population (30).

Previous studies have suggested that schizophrenia may offer some protection against cancer incidence, and the mechanisms of action may include, such as, the p53 gene producing, through apoptosis, the dual beneficial effects of disrupting neurodevelopment and reducing the risk of cancer (31);

It has also been shown that schizophrenia also increases the incidence of cancer, and the mechanism of action may be as follows, smoking rates among people with schizophrenia are typically twice as high as in the background population, implying a higher incidence of cancer among people with schizophrenia (32).

In summary, it is not possible to determine at the genetic level whether there is some association between schizophrenia and common cancers due to the presence of more confounding factors. In this case, it may not be appropriate to assess the effect of schizophrenia on cancer. Therefore, we considered schizophrenia as an exposure factor and applied two-sample MR analysis to explore its association with common cancers. A similar two-sample MR analysis conducted by *Yuan K et al.* found that schizophrenia is associated with an increased risk of breast, ovarian, and thyroid cancers (13), which is consistent with our own MR results, although there are some differences. In the present study, encouragingly, we found that schizophrenia increased the risk of lung cancer (OR= 1.0007; 95%CI, 1.0001-1.0013), colorectal cancer (OR = 1.0009; 95% CI, 1.0001-1.0018) and decreased the risk of malignant neoplasm of stomach (OR = 0.8502; 95% CI, 0.7230-0.9998);, in addition to increasing the risk of breast cancer (OR = 1.0011; CI, 1.0001-1.0022; *p* =0.0352), ovarian cancer (OR = 1.0770; CI, 1.0352-1.1203; *p* = 0.0002) and thyroid cancer(OR = 1.5482; CI, 1.1112-2.1569; *p* = 0.0098). Our study’s findings, which were obtained through MR analysis, may offer more reliable conclusions than previous studies that relied primarily on observational methods. This is because MR analysis is not subject to the influence of confounding factors or reverse causality.

This study has several limitations that require attention from an academic perspective. First, one such limitation is the difficulty in completely excluding the influence of potential directional pleiotropy in any MR study. However, it should be noted that evidence of pleiotropic effects was not observed in most MR-Egger intercept tests, except for colorectal cancer. Furthermore, similar results were observed in the sensitivity analyses. Second, the limitations of the examined GWASs include their focus on individuals of European ancestry, which may restrict the applicability of our findings to other ethnic groups. It is important to note that schizophrenia varies in terms of treatment options and tools across different regions, including Europe and other countries. However, the fact that the study population primarily consisted of individuals of European ancestry reduces the likelihood of population-stratification bias affecting our results. Third, the original GWAS study solely focused on individuals with schizophrenia, without specifying the type of schizophrenia. Unfortunately, we lacked specific information regarding the type and duration of the illness, which restricted our ability to perform additional analyses.

## 5 Conclusion

This two-sample MR analysis found a causal positive association between schizophrenia and breast, ovarian, thyroid, lung, and colorectal cancers, and a causal inverse association with gastric malignancies. No influences were observed for liver cell carcinoma, head and neck cancer, prostate cancer, esophageal cancer, and pancreatic cancer. For people with schizophrenia, cancer prevention is crucial.

## Data availability statement

The original contributions presented in the study are included in the article/**Supplementary Material**. Further inquiries can be directed to the corresponding author.

## Author contributions

XG: Investigation, Writing – review & editing, Supervision, Project administration. KZ: Writing – original draft, Conceptualization, Data curation. LZ: Project administration. NC: Funding acquisition, Writing – original draft. GH: Data curation, Software. GF: Formal analysis, Validation. QW: Software. XW: Formal analysis, Resources.
