# Supplementary material for: Causal associations between schizophrenia and cancers risk: a Mendelian randomization study
Source: Front Oncol. 2023 Nov 17;13:1258015. doi: 10.3389/fonc.2023.1258015 (PMC10693432; doi:10.3389/fonc.2023.1258015)
Supplement: Supplementary file 1 [file Table_1.doc]

Supplementary Table S1**.** Baseline characteristics of schizophrenia and cancer.

| Trait | Year | Author | Population | Sample Size | Ncase | Ncontrol | nSNP |
| --- | --- | --- | --- | --- | --- | --- | --- |
| Schizophrenia | 2022 | Trubetskoy V | Mixed | 320,404 | 76,755 | 243,649 | 7585420 |
| Lung cancer | 2021 | Burrows | European | 374,687 | 2,671 | 372,016 | 11,078,115 |
| Liver cell carcinoma | 2021 | Burrows | European | 372,184 | 168 | 372,016 | 6,304,034 |
| Thyroid cancer | 2013 | Kohler A | European | 1080 | 649 | 431 | 572028 |
| Colorectal cancer | 2021 | Burrows | European | 377,673 | 5657 | 372,016 | 11,738,639 |
| Ovarian cancer | 2017 | Phelan | European | 66,450 | 25,509 | 40,941 | - |
| Prostate cancer | 2021 | Burrows | European | 182,625 | 9,132 | 173,493 | - |
| Breast cancer | 2017 | Neale | European | 337,159 | 7,480 | 329,679 | 10,894,596 |
| Oesophageal cancer | 2021 | Burrows | European | 372,756 | 740 | 372,016 | 8,970,465 |
| Head and neck cancer | 2021 | Burrows | European | 373,122 | 1,106 | 372,016 | 9,655,080 |
| Malignant neoplasm of stomach | 2021 | - | European | - | 633 | 218,159 | 16,380,466 |
| Pancreatic cancer | 2009 | Amundadottir L | European | 3,835 | 1,896 | 1,939 | 521,863 |

Supplementary Table S2. Single nucleotide polymorphisms used as instrumental variables in the Mendelian randomization analyses of schizophrenia

| SNP | Chr | EA | OA | Beta | Se | Pval |
| --- | --- | --- | --- | --- | --- | --- |
| rs3900555 | 1 | A | G | 0.0428971 | 0.0075 | 9.95E-09 |
| rs10779702 | 1 | G | A | -0.0526968 | 0.0084 | 3.22E-10 |
| rs56335113 | 1 | G | A | -0.0653007 | 0.0081 | 1.06E-15 |
| rs6673880 | 1 | G | A | 0.0559991 | 0.0083 | 1.56E-11 |
| rs10157075 | 1 | T | A | -0.0590045 | 0.0101 | 5.26E-09 |
| rs12039854 | 1 | G | T | 0.0551005 | 0.0092 | 2.57E-09 |
| rs7515363 | 1 | T | C | -0.0491044 | 0.0083 | 3.43E-09 |
| rs61787564 | 1 | A | G | -0.064401 | 0.0113 | 1.34E-08 |
| rs12042444 | 1 | T | C | -0.0642979 | 0.0077 | 8.53E-17 |
| rs11587347 | 1 | G | C | 0.102299 | 0.0135 | 4.00E-14 |
| rs558120 | 1 | C | T | 0.0584981 | 0.0106 | 3.32E-08 |
| rs6588168 | 1 | T | C | 0.0458974 | 0.0075 | 1.17E-09 |
| rs11165846 | 1 | G | C | 0.0544034 | 0.0083 | 6.45E-11 |
| rs4950119 | 1 | C | A | 0.0906033 | 0.0102 | 8.36E-19 |
| rs12138231 | 1 | A | T | 0.0663001 | 0.0108 | 7.37E-10 |
| rs10559 | 1 | G | A | 0.0440982 | 0.008 | 4.02E-08 |
| rs60124939 | 1 | T | C | -0.052896 | 0.009 | 3.76E-09 |
| rs16851048 | 1 | C | T | 0.0680968 | 0.0091 | 8.28E-14 |
| rs1486472 | 1 | A | G | 0.0566021 | 0.0079 | 9.16E-13 |
| rs3770752 | 2 | G | A | -0.0592967 | 0.0084 | 1.74E-12 |
| rs62152282 | 2 | T | A | 0.0580953 | 0.0094 | 6.40E-10 |
| rs2167378 | 2 | T | C | -0.0577027 | 0.0075 | 9.90E-15 |
| rs6715416 | 2 | G | A | -0.0600033 | 0.0075 | 1.05E-15 |
| rs34181670 | 2 | C | T | -0.0485997 | 0.0085 | 1.13E-08 |
| rs999494 | 2 | T | C | -0.0567016 | 0.0096 | 3.62E-09 |
| rs62183855 | 2 | C | A | -0.061001 | 0.0102 | 1.97E-09 |
| rs10173857 | 2 | T | C | -0.0501036 | 0.0091 | 3.84E-08 |
| rs17247190 | 2 | G | A | 0.0517039 | 0.0092 | 2.15E-08 |
| rs1396728 | 2 | T | C | -0.0477992 | 0.0079 | 1.63E-09 |
| rs6546857 | 2 | G | A | 0.0542028 | 0.0094 | 8.70E-09 |
| rs6430491 | 2 | A | G | -0.0539956 | 0.0087 | 6.71E-10 |
| rs12991836 | 2 | C | A | 0.0555021 | 0.0078 | 8.19E-13 |
| rs60617652 | 2 | A | G | -0.0461963 | 0.0076 | 1.00E-09 |
| rs11680723 | 2 | G | C | 0.0842951 | 0.0102 | 1.05E-16 |
| rs11693094 | 2 | T | C | -0.0555009 | 0.0076 | 3.20E-13 |
| rs2033996 | 2 | A | T | 0.0519988 | 0.0095 | 4.33E-08 |
| rs68002929 | 2 | A | T | 0.0724954 | 0.0101 | 5.52E-13 |
| rs1881046 | 2 | T | G | -0.0443996 | 0.0079 | 1.92E-08 |
| rs13090130 | 3 | A | G | -0.052099 | 0.0077 | 1.04E-11 |
| rs11129383 | 3 | A | G | 0.0451961 | 0.0082 | 3.98E-08 |
| rs4678552 | 3 | A | G | 0.0538017 | 0.0086 | 3.19E-10 |
| rs167924 | 3 | G | A | 0.0540022 | 0.0085 | 1.84E-10 |
| rs10935184 | 3 | C | T | -0.0608975 | 0.0078 | 7.93E-15 |
| rs17194490 | 3 | T | G | 0.0763952 | 0.0113 | 1.53E-11 |
| rs1399551 | 3 | A | T | 0.0542978 | 0.0082 | 4.51E-11 |
| rs525297 | 3 | C | A | -0.0459958 | 0.0079 | 5.81E-09 |
| rs7647398 | 3 | T | C | -0.0828971 | 0.0098 | 2.46E-17 |
| rs6804677 | 3 | C | T | 0.0505988 | 0.0081 | 3.84E-10 |
| rs59971314 | 3 | C | G | 0.0460963 | 0.0079 | 5.88E-09 |
| rs59518103 | 4 | C | T | -0.0780993 | 0.0143 | 4.51E-08 |
| rs215483 | 4 | A | G | 0.0471021 | 0.008 | 4.23E-09 |
| rs61405217 | 4 | T | C | -0.0509972 | 0.0076 | 1.43E-11 |
| rs35734242 | 4 | C | T | 0.0513986 | 0.0077 | 2.24E-11 |
| rs28521069 | 4 | T | C | 0.0464 | 0.0079 | 4.69E-09 |
| rs1454606 | 4 | T | C | 0.0631954 | 0.0102 | 5.00E-10 |
| rs28454198 | 4 | C | G | -0.0477992 | 0.0077 | 5.97E-10 |
| rs28482891 | 4 | A | C | 0.0430954 | 0.0079 | 4.01E-08 |
| rs7681616 | 4 | A | C | -0.0489044 | 0.0088 | 2.70E-08 |
| rs12498839 | 4 | A | G | 0.117096 | 0.0149 | 3.24E-15 |
| rs13159353 | 5 | C | G | -0.0432989 | 0.0078 | 2.77E-08 |
| rs10117 | 5 | A | G | -0.052896 | 0.0079 | 1.88E-11 |
| rs10035564 | 5 | G | A | 0.0594953 | 0.0078 | 1.70E-14 |
| rs7701188 | 5 | A | G | -0.0746992 | 0.0118 | 2.81E-10 |
| rs252812 | 5 | G | A | -0.052896 | 0.0091 | 5.77E-09 |
| rs3733710 | 5 | T | C | -0.0448969 | 0.0077 | 5.52E-09 |
| rs7709645 | 5 | C | G | 0.0649973 | 0.0078 | 6.11E-17 |
| rs11241041 | 5 | T | G | 0.0596014 | 0.0096 | 5.35E-10 |
| rs72723227 | 5 | A | G | -0.0503033 | 0.008 | 3.76E-10 |
| rs11740474 | 5 | T | A | 0.0484027 | 0.0082 | 4.43E-09 |
| rs12652777 | 5 | C | T | -0.0443996 | 0.0076 | 5.10E-09 |
| rs9687282 | 5 | G | T | 0.0475949 | 0.0082 | 7.34E-09 |
| rs72802887 | 5 | G | A | -0.066602 | 0.0093 | 8.53E-13 |
| rs9470670 | 6 | G | T | 0.0555973 | 0.0099 | 1.91E-08 |
| rs2206956 | 6 | A | G | 0.0447045 | 0.0076 | 4.04E-09 |
| rs34555420 | 6 | T | G | -0.168696 | 0.0173 | 1.82E-22 |
| rs6938026 | 6 | G | A | -0.0603046 | 0.0088 | 7.23E-12 |
| rs6925079 | 6 | C | T | 0.0465048 | 0.0077 | 1.81E-09 |
| rs80249955 | 6 | T | C | 0.154597 | 0.0283 | 4.63E-08 |
| rs9487653 | 6 | G | A | -0.0553969 | 0.0101 | 4.40E-08 |
| rs9459170 | 6 | C | T | -0.0511018 | 0.0093 | 3.83E-08 |
| rs55648125 | 6 | G | A | 0.072001 | 0.0132 | 4.42E-08 |
| rs217310 | 6 | T | A | -0.0487044 | 0.0075 | 9.94E-11 |
| rs12190758 | 6 | G | A | 0.0613011 | 0.0094 | 6.01E-11 |
| rs9398171 | 6 | T | C | -0.0503033 | 0.0081 | 4.48E-10 |
| rs58120505 | 7 | C | T | -0.0825012 | 0.0075 | 6.26E-28 |
| rs6946576 | 7 | A | G | -0.0456997 | 0.0084 | 4.94E-08 |
| rs13233308 | 7 | T | C | -0.0458048 | 0.0075 | 1.23E-09 |
| rs7803571 | 7 | T | C | -0.0575989 | 0.0076 | 4.49E-14 |
| rs12671608 | 7 | C | T | 0.0598986 | 0.0109 | 4.44E-08 |
| rs2944821 | 7 | C | G | -0.0493995 | 0.0078 | 2.51E-10 |
| rs1593304 | 7 | G | A | 0.0631954 | 0.0097 | 5.91E-11 |
| rs7779548 | 7 | A | G | -0.0712969 | 0.0079 | 1.82E-19 |
| rs35274762 | 7 | C | T | -0.102304 | 0.0122 | 5.91E-17 |
| rs56226048 | 7 | T | G | 0.0605996 | 0.0109 | 3.09E-08 |
| rs2349487 | 7 | T | C | 0.0456042 | 0.0078 | 5.71E-09 |
| rs79210963 | 7 | C | T | 0.0842951 | 0.0126 | 1.86E-11 |
| rs2470951 | 7 | T | A | 0.0527997 | 0.0075 | 2.72E-12 |
| rs211824 | 7 | A | G | -0.0481042 | 0.0077 | 4.51E-10 |
| rs73229090 | 8 | A | C | -0.097798 | 0.014 | 2.44E-12 |
| rs74804370 | 8 | C | T | 0.119798 | 0.0218 | 3.68E-08 |
| rs1434281 | 8 | A | G | -0.045499 | 0.008 | 1.08E-08 |
| rs4129585 | 8 | C | A | -0.065104 | 0.0077 | 2.51E-17 |
| rs59498392 | 8 | G | C | -0.0574006 | 0.0089 | 1.10E-10 |
| rs11987861 | 8 | G | A | -0.0481042 | 0.0086 | 1.97E-08 |
| rs6471815 | 8 | G | A | -0.050503 | 0.0076 | 2.71E-11 |
| rs10957321 | 8 | A | G | 0.0490012 | 0.0074 | 3.67E-11 |
| rs11779128 | 8 | T | C | -0.0425038 | 0.0077 | 3.98E-08 |
| rs10103330 | 8 | A | T | 0.0628014 | 0.0093 | 1.45E-11 |
| rs73219806 | 8 | A | C | 0.071496 | 0.0094 | 2.71E-14 |
| rs4043663 | 8 | A | G | -0.0575045 | 0.0087 | 3.63E-11 |
| rs10086619 | 8 | G | A | 0.0615988 | 0.0098 | 2.97E-10 |
| rs498591 | 9 | T | A | 0.0594953 | 0.0105 | 1.36E-08 |
| rs505061 | 9 | A | C | 0.0499994 | 0.0074 | 1.29E-11 |
| rs10985811 | 9 | C | T | 0.0532953 | 0.0093 | 9.33E-09 |
| rs6479494 | 9 | G | A | 0.0584981 | 0.0102 | 9.57E-09 |
| rs13293831 | 9 | T | C | 0.0591027 | 0.0103 | 8.73E-09 |
| rs2381411 | 9 | C | T | 0.0428031 | 0.0077 | 2.42E-08 |
| rs4339716 | 9 | G | T | 0.0530949 | 0.0097 | 4.03E-08 |
| rs72761691 | 9 | C | A | 0.0618967 | 0.0111 | 2.56E-08 |
| rs61857878 | 10 | T | A | -0.0611045 | 0.0096 | 1.84E-10 |
| rs7902292 | 10 | C | T | -0.0499038 | 0.0091 | 4.43E-08 |
| rs113899647 | 10 | T | C | 0.0898044 | 0.0163 | 3.90E-08 |
| rs7893279 | 10 | G | T | -0.0995014 | 0.0123 | 6.54E-16 |
| rs12571643 | 10 | A | G | -0.113498 | 0.0113 | 1.25E-23 |
| rs17731 | 10 | A | G | 0.0589011 | 0.0077 | 2.95E-14 |
| rs2279311 | 10 | C | T | 0.048004 | 0.0088 | 4.12E-08 |
| rs12363019 | 11 | A | T | 0.0523992 | 0.008 | 5.91E-11 |
| rs708228 | 11 | T | C | 0.0488017 | 0.0083 | 3.99E-09 |
| rs3016382 | 11 | A | C | -0.0459003 | 0.0076 | 1.34E-09 |
| rs10767735 | 11 | C | G | -0.0503984 | 0.0077 | 7.15E-11 |
| rs2902858 | 11 | C | T | 0.0880992 | 0.0101 | 2.69E-18 |
| rs72943392 | 11 | C | G | 0.0499048 | 0.0091 | 4.71E-08 |
| rs77502336 | 11 | C | G | 0.054604 | 0.008 | 6.94E-12 |
| rs11219774 | 11 | C | A | -0.0690969 | 0.0101 | 7.79E-12 |
| rs61902811 | 11 | A | G | -0.0674998 | 0.0086 | 5.33E-15 |
| rs11222406 | 11 | G | A | -0.0407004 | 0.0074 | 3.65E-08 |
| rs4936216 | 11 | T | C | -0.0773036 | 0.0103 | 6.28E-14 |
| rs3017989 | 11 | C | T | 0.0473956 | 0.0078 | 1.37E-09 |
| rs302317 | 12 | A | G | -0.0524027 | 0.0081 | 7.87E-11 |
| rs61920311 | 12 | C | A | -0.0459958 | 0.008 | 8.70E-09 |
| rs578470 | 12 | C | T | 0.0456984 | 0.0079 | 8.42E-09 |
| rs4766428 | 12 | T | C | 0.0680005 | 0.0082 | 8.11E-17 |
| rs61937595 | 12 | T | C | -0.119799 | 0.0156 | 1.53E-14 |
| rs2686386 | 12 | T | C | -0.0546965 | 0.0094 | 6.28E-09 |
| rs1790135 | 12 | T | C | -0.0842033 | 0.0084 | 6.91E-24 |
| rs4298967 | 12 | G | A | -0.0825012 | 0.0086 | 1.28E-21 |
| rs1526803 | 12 | G | C | -0.0465018 | 0.0082 | 1.66E-08 |
| rs61924144 | 12 | C | A | 0.0785996 | 0.0134 | 4.15E-09 |
| rs6538539 | 12 | T | G | -0.0450976 | 0.0074 | 9.13E-10 |
| rs1426371 | 12 | A | G | 0.0515038 | 0.0087 | 2.67E-09 |
| rs11619756 | 13 | A | G | -0.0507977 | 0.008 | 2.04E-10 |
| rs9569795 | 13 | C | T | 0.0635044 | 0.0102 | 5.54E-10 |
| rs9597388 | 13 | A | G | -0.0625991 | 0.0097 | 8.98E-11 |
| rs9545047 | 13 | C | A | -0.0551035 | 0.0079 | 4.12E-12 |
| rs61973697 | 13 | A | G | 0.0548998 | 0.0097 | 1.57E-08 |
| rs650520 | 13 | T | G | 0.0471964 | 0.0081 | 5.65E-09 |
| rs12877581 | 13 | C | G | 0.0515038 | 0.0092 | 2.44E-08 |
| rs10148671 | 14 | C | T | 0.0467038 | 0.0079 | 4.14E-09 |
| rs12883788 | 14 | T | C | 0.0542028 | 0.0076 | 1.30E-12 |
| rs1676062 | 14 | A | C | -0.0446961 | 0.0078 | 1.02E-08 |
| rs10873538 | 14 | G | T | 0.0593997 | 0.0079 | 6.14E-14 |
| rs1540840 | 14 | C | G | -0.0569 | 0.0088 | 9.65E-11 |
| rs2190873 | 14 | C | T | -0.0628997 | 0.0074 | 2.12E-17 |
| rs11632947 | 15 | T | C | 0.0553964 | 0.0075 | 1.64E-13 |
| rs637137 | 15 | A | T | -0.0653007 | 0.0084 | 8.31E-15 |
| rs56282503 | 15 | C | T | 0.055703 | 0.009 | 6.63E-10 |
| rs2929278 | 15 | T | C | -0.0569 | 0.0087 | 6.01E-11 |
| rs11854073 | 15 | A | G | -0.0479994 | 0.0081 | 3.66E-09 |
| rs10906984 | 15 | C | A | -0.0491044 | 0.0076 | 8.04E-11 |
| rs176648 | 15 | G | T | -0.0461963 | 0.0076 | 1.39E-09 |
| rs4702 | 15 | A | G | -0.078996 | 0.0079 | 1.76E-23 |
| rs9925915 | 16 | C | G | -0.057004 | 0.0075 | 2.51E-14 |
| rs11862968 | 16 | G | C | 0.054604 | 0.0093 | 4.71E-09 |
| rs12925872 | 16 | T | C | 0.067305 | 0.0091 | 1.87E-13 |
| rs11076631 | 16 | G | A | -0.050503 | 0.0083 | 1.33E-09 |
| rs8048039 | 16 | T | A | -0.0511968 | 0.0081 | 1.98E-10 |
| rs9302397 | 16 | A | G | 0.0488017 | 0.0086 | 1.49E-08 |
| rs11647188 | 16 | G | A | -0.0441987 | 0.0077 | 1.08E-08 |
| rs12950148 | 17 | G | A | 0.0561049 | 0.01 | 1.92E-08 |
| rs959071 | 17 | T | C | -0.075497 | 0.0109 | 4.05E-12 |
| rs4793888 | 17 | A | G | 0.0573961 | 0.0094 | 1.12E-09 |
| rs4293 | 17 | A | G | 0.0428031 | 0.0076 | 2.21E-08 |
| rs11263770 | 17 | A | G | -0.0425996 | 0.0076 | 1.74E-08 |
| rs2696466 | 17 | G | A | -0.0522983 | 0.0081 | 1.01E-10 |
| rs12943566 | 17 | G | A | 0.0486967 | 0.0079 | 7.85E-10 |
| rs75329315 | 17 | G | T | -0.079698 | 0.013 | 9.38E-10 |
| rs17512480 | 18 | A | T | 0.174603 | 0.0242 | 5.60E-13 |
| rs7238071 | 18 | G | A | 0.0591027 | 0.0081 | 2.48E-13 |
| rs35360904 | 18 | G | T | -0.0562007 | 0.0075 | 5.40E-14 |
| rs11083369 | 18 | T | G | 0.0608015 | 0.0083 | 3.05E-13 |
| rs715170 | 18 | T | C | -0.061001 | 0.0088 | 5.11E-12 |
| rs72980087 | 18 | A | G | 0.0610034 | 0.0077 | 1.92E-15 |
| rs72986630 | 19 | T | C | 0.107797 | 0.0174 | 6.31E-10 |
| rs322124 | 19 | G | C | 0.0521041 | 0.0089 | 4.53E-09 |
| rs8101499 | 19 | G | A | -0.0592024 | 0.0077 | 1.77E-14 |
| rs3810450 | 19 | C | T | -0.0892006 | 0.0157 | 1.37E-08 |
| rs7251 | 19 | G | C | -0.0582029 | 0.008 | 3.79E-13 |
| rs758749 | 19 | T | C | 0.0617052 | 0.0109 | 1.49E-08 |
| rs2387414 | 19 | C | G | 0.0488962 | 0.0081 | 1.40E-09 |
| rs1006945 | 20 | T | G | 0.0601004 | 0.0074 | 3.30E-16 |
| rs11696755 | 20 | C | T | 0.0631954 | 0.01 | 2.61E-10 |
| rs8134737 | 21 | T | A | 0.0467038 | 0.0076 | 9.48E-10 |
| rs229362 | 21 | A | G | -0.0531047 | 0.0095 | 2.32E-08 |
| rs1058167 | 22 | G | A | 0.0544034 | 0.0083 | 4.89E-11 |
| rs732381 | 22 | T | A | 0.065798 | 0.0089 | 1.17E-13 |
| rs6010045 | 22 | C | T | 0.0458031 | 0.0081 | 1.60E-08 |
