# Supplementary material for: Causal associations between schizophrenia and cancers risk: a Mendelian randomization study
Source: Front Oncol. 2023 Nov 17;13:1258015. doi: 10.3389/fonc.2023.1258015 (PMC10693432; doi:10.3389/fonc.2023.1258015)
Supplement: Supplementary file 2 [file DataSheet_1.doc]

**Supplementary Figure Captions**

**Supplementary Figure S1. Scatter plot of the association of schizophrenia** **with cancer.**

A: Lung cancer; B: Liver cell carcinoma; C: Thyroid cancer; D: Colorectal cancer; E: Ovarian cancer; F: Prostate cancer; G: Breast cancer; H: Oesophageal cancer; I: Head and neck cancer; J: Malignant neoplasm of stomach; K: Pancreatic cancer

Each black dot indicates a SNP, plotted by the estimate of SNP on individual schizophrenia and the estimate of SNP on the risk of cancer with standard error bars. The slopes of the lines correspond to causal estimates using each of the different methods.

SNP: single nucleotide polymorphism

**Supplementary Figure S2. Forest plot of the association of schizophrenia** **with cancer.**

A: Lung cancer; B: Liver cell carcinoma; C: Thyroid cancer; D: Colorectal cancer; E: Ovarian cancer; F: Prostate cancer; G: Breast cancer; H: Oesophageal cancer; I: Head and neck cancer; J: Malignant neoplasm of stomach; K: Pancreatic cancer

The dot and bar indicate the causal estimate of schizophrenia on risks of cancer.

**Supplementary Figure S3. Leave-one-out sensitivity analysis of the association of schizophrenia** **with cancer.**

A: Lung cancer; B: Liver cell carcinoma; C: Thyroid cancer; D: Colorectal cancer; E: Ovarian cancer; F: Prostate cancer; G: Breast cancer; H: Oesophageal cancer; I: Head and neck cancer; J: Malignant neoplasm of stomach; K: Pancreatic cancer

The dot and bar indicate the estimates and 95% confidence interval when the specific single nucleotide polymorphism is removed.

**Supplementary Figure S4. Funnel plot of the association of of schizophrenia** **with cancer.**

A: Lung cancer; B: Liver cell carcinoma; C: Thyroid cancer; D: Colorectal cancer; E: Ovarian cancer; F: Prostate cancer; G: Breast cancer; H: Oesophageal cancer; I: Head and neck cancer; J: Malignant neoplasm of stomach; K: Pancreatic cancer

Each black dot indicates a single nucleotide polymorphism.
